# Multidisciplinary Assessment and Diagnosis of Conversion Disorder in a Patient with Foreign Accent Syndrome

**DOI:** 10.3233/BEN-2011-0332

**Published:** 2011-08-29

**Authors:** Harrison N. Jones, Tyler J. Story, Timothy A. Collins, Daniel DeJoy, Christopher L. Edwards

**Affiliations:** ^1^Department of SurgeryDivision of Speech Pathology and AudiologyDuke University Medical CenterNCUSA; ^2^Department of MedicineDivision of NeurologyDuke University Medical CenterNCUSA; ^3^Department of CommunicationsNorth Carolina State UniversityDuke University Medical CenterNCUSA; ^4^Department of PsychiatryDivision of Medical PsychologyDuke University Medical CenterNCUSA; ^5^Department of MedicineDivision of HematologyDuke University Medical CenterNCUSA

**Keywords:** Foreign accent syndrome, conversion disorder, neuropsychological assessment, case study, speech disorders

## Abstract

Multiple reports have described patients with disordered articulation and prosody, often following acute aphasia, dysarthria, or apraxia of speech, which results in the perception by listeners of a foreign-like accent. These features led to the term foreign accent syndrome (FAS), a speech disorder with perceptual features that suggest an indistinct, non-native speaking accent. Also correctly known as psuedoforeign accent, the speech does not typically match a specific foreign accent, but is rather a constellation of speech features that result in the perception of a foreign accent by listeners. The primary etiologies of FAS are cerebrovascular accidents or traumatic brain injuries which affect cortical and subcortical regions critical to expressive speech and language production. Far fewer cases of FAS associated with psychiatric conditions have been reported. We will present the clinical history, neurological examination, neuropsychological assessment, cognitive-behavioral and biofeedback assessments, and motor speech examination of a patient with FAS without a known vascular, traumatic, or infectious precipitant. Repeated multidisciplinary examinations of this patient provided convergent evidence in support of FAS secondary to conversion disorder. We discuss these findings and their implications for evaluation and treatment of rare neurological and psychiatric conditions.

## 1. Introduction

Foreign accent syndrome (FAS) is a rarely encountered speech disorder that presents with abnormalities in articulation and prosody which are perceived by listeners as sounding similar to a foreign accent. Traditionally, FAS is most commonly associated with left-hemisphere lesions [1] secondary to CVA [2–13] and traumatic brain injury (TBI) [14–17]. The condition is often preceded or accompanied by other communication disorders such as aphasia, dysarthria, and apraxia of speech [18]. More recently, FAS has been associated with a variety of neurological illnesses, including multiple sclerosis [19,20], primary progressive aphasia [21], cerebellar hypoperfusion [22], and metastatic breast cancer [23]. Several case reports of patients with FAS and psychiatric illness without a known brain lesion have also appeared, including patients with bipolar disease [24], psychosis [25,26], psychogenic illness [27], and conversion disorder [28,29]. In this paper, we describe the multidisciplinary assessment and diagnosis of FAS secondary to conversion disorder.

ISSN 0953-4180/11/$27.50 © 2011 – IOS Press and the authors. All rights reserved

## 2. Patient description

The patient was a 39-year-old, right-handed African-American woman, born and raised in the southeastern region of the United States. She described an unremarkable birth and developmental trajectory with no history of a learning disorder or behavioral problems during childhood. She obtained her high-school diploma without academic incident and worked in childcare prior to the onset of chronic daily headaches. She denied a history of emotional, physical, or sexual abuse. She also denied notable use of alcohol, tobacco, or illicit substances. During the course of her treatment, she initiated the process of application for long-term disability and did not return to work during her treatment or follow-up. Although not working, there were few if any functional impairments noted by her evaluation and treatment team or described by the patient.

She presented to the emergency department (ED) of an academic medical center for treatment of persistent headache, decreased right-sided sensation, gait disturbance, and disruption of speech articulation and prosody. She presented to the ED approximately three months after the onset of headaches she described as a severe, throbbing pain in the right temporal-parietal region intermittently accompanied by nausea, vomiting, photophobia, and phonophobia. She reported at that visit having "multiple" evaluations at other hospitals for the same complaints. Upon presentation, she reported onset of her sensory, speech, and gait symptoms three to four days prior to seeking medical attention at our ED. She was taking ibuprofen at the time of admission and reported allergic responses to dextropropoxyphene, hydrocodone, and naproxen. She was evaluated in the ED with a CT scan of the brain, and treated with sumatriptan for her migraine with some improvement. She was scheduled to follow-up with neurology on an outpatient basis and discharged home.

## 3. Neurological examination

The patient was first seen in the neurology clinic approximately 1 month after her ED visit. The patient and her family reported previous assessments in multiple facilities with unremarkable brain imaging results. Although she reported improvement, she discontinued sumatriptan as it reportedly caused her to feel ill and was associated with chest discomfort. She described only two days over the prior four months prior to neurology assessment as headache free. Examination in the neurology clinic on her initial visit found inconsistent sensory examination with features of non-physiologic sensory loss including "splitting the midline". Motor examination found inconsistencies, with normal strength observed in all extremities during portions of the clinic visit and effort dependant weakness during formal motor testing of the extremities.

Over the course of two years, she was evaluated in the neurology clinic eleven times. General physical examination was repeatedly normal, as were cranial nerve examinations and tendon reflexes. Markedly variable weakness, which generally appeared effort dependent, in one or both lower extremities and the right upper extremity, was repeatedly exhibited. Strength testing during formal examination and observed physical behavior in clinic frequently were inconsistent. For example, no effort would be exhibited during formal motor testing of the right leg, yet the ability to use the right leg to step off the exam table and bear weight when walking was easily observed. The patient's gait revealed the following features which have been found to be associated with psychogenic gait disorders: exaggerated effort, gait fluctuations, uneconomic postures, and dramatic give way weakness [30]. There was no evidence of hemiparesis. Finger nose finger and heel knee shin testing were preserved without evidence of ataxia. There were no abnormal spontaneous movements and muscle tone was normal. Additional evidence for the presence of a psychogenic gait disorder was provided by the presence of a positive "chair test" as described by Okun and colleagues [31]. This test requires both walking and self-propelling in a swivel chair with wheels and the results are compared. Okun et al. reported that improved performance on the chair versus walking, or a positive "chair test", was suggestive of a psychogenic gait disorder.

She reported episodes of memory loss and "blackouts". History from the patient and family regarding the "blackouts" were suboptimal, but it was found that the patient had preserved consciousness, and appeared to be cognitively normal during these events, consistent with a fugue event. There was no history elicited of seizure activity or tonic clonic activity

Speech was produced initiated and fluently with appropriate content. She demonstrated the ability to answer questions, name objects, repeat words and sentences, and verbally express herself in connected speech. When asked questions regarding her speech difficulty, she offered spontaneous responses that were organized, grammatically correct, and pragmatically appropriate. No phonemic or semantic paraphasias were present. Additionally, she was able to follow commands and comprehend conversational speech. Although a foreign-sounding accent was present and she had a slow rate of speech with mild distortions and prosodic abnormalities, her speech was not characteristic of a particular dysarthria type(s) and associated neuromotor deficits (e.g., spasticity, incoordination) known to accompany neurologic disease and manifest upon the speech mechanism. Not surprisingly in a condition also appropriately known as pseudoforeign accent, listeners could not agree on the "origin" of the accent though a Jamaican accent was most commonly described. Her speech accent varied over time though FAS always persisted to some degree. She completed a pain scale rating during each neurology visit as this was her primary complaint. Her pain scale rating ranged from 4 to 9 on an equal-appearing interval scale of 0-10 (i.e., 0 = no pain; 10 = worst pain ever) and over multiple visits, it was noted that her pain scale rating did not correlate with the severity of her physical findings on exam. Her gait disorder and "foreign accent" varied with no perceived relationship to her reported pain complaints, though she reported that the severity of her headache influenced how affected her speech was. CT of the head, MRI of the brain with and without contrast, MRI of the cervical spine, and EEG results were unremarkable. Her neurologic presentation was considered most consistent with a functional disorder.

## 4. Neuropsychological assessment

Approximately 18 months after symptom onset, she was referred for a neuropsychological assessment. On the day of assessment, positive and appropriate affect was exhibited with no outward signs of a current mood disorder. She did not show observable signs of pain or discomfort. Thought processes were logical and linear, and she denied suicidal, homicidal, or paranoid content. She was pleasant and cooperative throughout testing.

She described difficulty with memory, concentration, judgment, reasoning, and word-finding. The patient and her husband stated that these symptoms started with the onset of headache and varied in severity depending on the intensity of her pain. Severe headaches were also associated with intensified speech disruption and agraphia, though she did not exhibit agraphia on the day of her evaluation. She described an episode approximately three months prior when she was unable to remember the names of family members for four days.

She estimated that she slept approximately six hours per night when taking zolpidem, though she also described several occasions of insomnia lasting three or more days due to severe cephalagia. The patient denied symptoms of depression or anxiety. A 30-pound weight loss over the preceding two months was reported, which she attributed to nausea secondary to headache.

A comprehensive neuropsychological examination using standardized instruments to address all major cognitive domains was administered. Specific measures by domain included: general intellectual function – Wechsler Adult Intelligence Scale-3rd Edition [WAIS-III, selected subtests; 32]; reading achievement and estimated premorbid ability – Wide Range Achievement Test [WRAT, 33]; executive function, processing speed, working memory, and attention – Trail Making Test [34], Stroop Color and Word Test [35], Ruff Figural Fluency [36], and WAIS-III subtests; memory – California Verbal Learning Test-2nd Edition [37], Wechsler Memory Scale-3rd Edition [38], and Brief Visuospatial Memory Test-Revised [BVMT-R, 39]; language function – Boston Naming Test [40], Multilingual Aphasia Exam [41], and categorical fluency (i.e., animal naming); visual-constructional skills – WAIS-III Block Design and Brief Visuospatial Memory Test -Revised (BVMT-R) figure copy; fine motor speed – Finger Tapping, Grooved Pegboard; effort and test engagement – Green Word Memory Test [42], Test of Memory Malingering [43]; and mood – Beck Depression Inventory-II [44].

Raw test scores and performance categories based on normative data are presented in Table 1. Her test results revealed severe impairments in several cognitive domains including memory, executive functions, language, and fine-motor skills. More subtle deficits were present on tests of attention, working memory, and auditory comprehension. In contrast, letter fluency, category fluency, confrontation naming, and nonverbal recall were within normal limits.

While test results suggested the presence of a severe cognitive disorder, there were concerns regarding the validity of the exam. Effort testing fell below expectation for immediate recognition (IR = 87.5), delayed recognition (DR = 77.5), and consistency (CNS = 70.0) on the Green Word Memory Test, a forced-choice word recognition procedure developed to assess effort and engagement in neuropsychological procedures. Her exam was also notable for variability in performance inconsistent with task difficulty. For example, simple auditory comprehension fell below expectation, while comprehension of more difficult, multi-

Table 1
Neuropsychological assessment data

| Category | Test | Scaled scores | Description |
|---|---|---|---|
| Premorbid Estimate | WRAT Reading | 46 | Average (high school) |
| Intellectual Functioning | WAIS-III | | |
| | FSIQ | 65 | Impaired |
| | VIQ | 76 | Borderline Impaired |
| | PIQ | 60 | Impaired |
| | WMI | 71 | Borderline Impaired |
| | PSI | 69 | Impaired |
| Executive Functions | Trail Making Test | | |
| | Trails A | 146" | Severely Impaired |
| | Trails B | 239", 1 error | Impaired |
| | Stroop Color and Word Test | | |
| | Word | 32 | Severely Impaired |
| | Color | 9 | Severely Impaired |
| | Color/Word | 27 | Impaired |
| | Ruff Figural Fluency Test | | |
| | Designs | 25 | Impaired |
| | Perseverative Errors | 0 | High Average |
| | WAIS-III | | |
| | Digit Span | 10 | Borderline Impaired |
| | Similarities | 16 | Borderline Impaired |
| | Matrix Reasoning | 4 | Impaired |
| Memory | WMS-III | | |
| | Logical Memory-I (LM) | 15 | Severely Impaired |
| | LM-II | 9 | Borderline Impaired |
| | Faces-I | 36 | Average |
| | Faces-II | 36 | Average |
| | California Verbal Learning Test-II | | |
| | Total | 30 | Impaired |
| | Immediate Free Recall | 5 | Impaired |
| | Immediate Cued Recall | 11 | Low Average |
| | Delayed Free Recall | 9 | Borderline Impaired |
| | Delayed Cued Recall | 11 | Low Average |
| | Recognition | 11 | Impaired |
| | *BVMT-R* | | |
| | Total | 22 | Low Average |
| | Delayed | 6 | Impaired |
| | Recognition Hits | 5 | Borderline/Low Average |
| Language | | | |
| | Boston Naming Test | 41 | Low Average |
| | COWA 'FAS' | 35 | Average |
| | Animal Naming | 12 | Low Average |
| | Multilingual Aphasia Exam | | |
| | Repetition | 5 | Impaired |
| | Auditory Comprehension | 15 | Borderline Impaired |
| | Token Test | 40 | Low Average |
| | Reading Comprehension | 16 | Borderline |
| Visuospatial | WAIS-III | | |
| | Block Design | 19 | Borderline Impaired |
| | BVMT-R | | |
| | Copy | 12/12 | Within normal limits |
| Sensorimotor | Grooved Pegboard | | |
| | dominant hand | 149, 1 error | Impaired |
| | nondominant hand | 130 | Impaired |
| | Finger Tapping | | |
| | dominant hand | 11.8 | Severely Impaired |
| | nondominant hand | 21.4 | Severely Impaired |
| Effort and Motivation | TOMM | | |
| | Trial 1 | 46 | Within normal limits |
| | Trial 2 | 47 | Within normal limits |
| | Green Word Memory Test | | |
| | Immediate | 87.5 | Caution |
| | Delayed | 77.5 | Failed |
| | Consistency | 70.0 | Failed |
| Mood | Beck Depression Inventory-2 | 13 | Subclinical |

Legend: WRAT Reading: Wide Range Achievement Test-Reading; WAIS-III: Wechsler Adult Intelligence Scale-3rd Edition; FSIQ: Full Scale Intellectual Quotient; VIQ: Verbal Intellectual Quotient; PIQ: Performance Intellectual Quotient; WMI: Working Memory Index; PSI: Processing Speed Index; WMS-III: Wechsler Memory Scale-3rd Edition; BVMT-R: Brief Visuospatial Memory Test-Revised; COWA 'FAS': Controlled Oral Word Association Test, Version FAS; TOMM: Test of Memory Malingering.

step directions was within normal limits. Additionally, cued verbal recall on a list-learning task was reasonably intact, while performance on a subsequent recognition trial was severely impaired. Finger-tapping speed also fell in the severely impaired range with the patient's raw score falling below criteria often used to verify adequate effort [45].

## 5. Cognitive-behavioral and biofeedback assessment

Following referral, she was seen for an initial assessment followed by 13 sessions of behavioral intervention over an 18-month period. Initial biofeedback assessment included psychological testing to determine the patient's suitability for biofeedback and to assist in the psychiatric diagnostic process. A diagnostic clinical interview, the Minnesota Multiphasic Personality Inventory, Second Edition (MMPI-2) [46], the Neuroticism Extroversion Openness Personality Inventory, Revised (NEO-PI-R) [47,48], the Symptoms Checklist, 90-items, Revised (SCL-90-R) [49], the BDI [44], and State Trait Anxiety Inventory (STAI) [50] were all utilized to assess the patient's psychiatric status. In interview, the patient described a "severe, continuous throbbing headache" concentrated on the right-side of her head which produced nausea. She also reported severe, chronic lower back pain. Her medical history was significant for memory problems, "black outs", and FAS onset around the time of her headaches. Family medical history was reportedly significant for diabetes, cancer, obesity, Alzheimer's disease (maternal grandparent), and headache.

Her responses to the validity items of the MMPI-2 revealed questionable validity (F-K = 16) and results were thus interpreted with caution. Clinical scales revealed a 1–3/3–1 code-type with a pronounced conversion-V profile. Individuals with this pattern of responses typically present with significant somatic concerns which are exacerbated in proportion to the magnitude of stressors (Scale 1 T = 80; Scale 2 T = 59; Scale 3 T = 87). Somatic complaints may be used as a proxy for the magnitude of psychological distress [46]. Patients with conversion disorder may manage emotional distress by converting negative emotional reactions and feelings into physical complaints that are interpreted as more socially acceptable. Such individuals often present as confused and disorganized.

Consistent with suspected conversion disorder, she scored in the "very low" range on the Neuroticism scale of the NEO-PI-R. Notably, this scale evaluates negative emotional symptoms associated with anxiety, anger and hostility, depression, self-consciousness, impulsiveness, and vulnerability. She also scored in the Average range on the Extraversion, Agreeableness, and Conscientiousness scales.

Responses on the SCL-90-R, a general checklist of psychiatric symptoms, produced a single clinical elevation ($T \geqslant 65$) on the Somatization scale ($T = 65$) indicating her endorsement of numerous, distressing physical symptoms with little emotional concern. Consistent with previous results, she did not elevate scales which typically reflect emotional distress. On the STAI, scores of 35 for state anxiety and 38 for trait anxiety were obtained, both of which are two standard deviations below the mean for reported anxiety in her age/sex peer group. Lastly, she scored below the clinical range for symptoms of depression on the BDI-2 (BDI-2 = 11). She strongly endorsed physical symptoms, such as appetite disturbance, but denied those associated with emotional distress, such as sadness, anhedonia, guilt, and irritation. Overall, the clinical interview and results of testing were most consistent with an Axis I psychiatric diagnosis of conversion disorder with mixed presentation.

## 6. Motor speech examination

Twenty months after her initial presentation to the ED, she was seen for a motor speech examination. The exam included a clinical interview regarding the nature and history of her speech problems and examination of the speech mechanism during a variety of tasks. The speech mechanism comprises the *structures* of the respiratory mechanism, larynx, velopharynx, and orofacial mechanism, also commonly referred to as the *processes* of respiration, phonation, resonance, and articulation, respectively. Prosody of speech is often considered a fifth speech process, though it is best conceptualized as comprising multiple speech features (e.g., intonation, stress, rate, rhythm) which result from the complex interactions of the structures of the speech mechanism.

The clinical interview obtained during this exam appeared to reveal some new or different details regarding her medical history, particularly as it pertained to her communication. She described the acute onset of severe headache approximately two years prior to evaluation, which confined her to bed for two or three days. It was difficult to determine the patient's communication status at this time, but she endorsed the notion that she likely made limited attempts to communicate due to the severity of her headache. However, this was followed by a period of two or three days of complete mutism, then the onset of speaking in what others perceived as a “foreign accent” which she did not notice until it was brought to her attention. The patient denied a history of travel out of the United States and had rarely left her home state. She reported limited exposure to individuals who spoke with a foreign accent and denied any substantial experience with a second language. Neither her speech change nor her headache had ever resolved, though the severity of both were described as variable.

The motor speech examination consisted of auditory-perceptual and instrumental measurement approaches. Tasks for auditory-perpetual speech assessment included high-effort nonspeech and speech tasks [51], repetition of words and sentences, reading of a standard passage (e.g., “The Grandfather Passage”), and elicitation of connected speech. Quantitative measurement approaches included lingual strength testing with a single use, air-filled intraoral silicone tongue bulb and the Iowa Oral Performance Instrument (IOPI; IOPI Medical LLC) and measurement of maximum inspiratory and expiratory pressure (i.e., strength) with a respiratory pressure meter (RPM; RPM01, Micro-Direct). Results of the speech examination follow:

### *6.1. Respiratory mechanism*

Assessment of the respiratory mechanism revealed a brisk sniff and a rapid, coordinated pant suggesting normal inspiratory strength and intact ability to complete a relatively simple, maximum performance respiratory task, highly dependent on respiratory muscle control and coordination of both the inspiratory and expiratory muscle systems. During isolated testing, her maximum loudness and range of loudness was reduced, though loudness in connected speech was within normal limits. Respiratory strength was measured using the RPM. Maximum inspiratory pressure (MIP) and maximum expiratory pressure (MEP) were obtained over three trials and the mean was obtained. MIP was 9 cm H20 (11, 7, 9; $sd = 2$) and MEP was 17 cm H20 (17, 19, 16; $sd = 1.53$). Normative data from Chen and Kuo in 160 healthy participants age 18–65, including 20 females 30–39 years old, are useful to interpret these findings [58]. In the 20 healthy females with a mean age of 35.8 years studied by Chen and Kuo, mean MIP was 76.0 cm H20 ($sd = 15.3$) and MEP was 92.8 cm H20 ($sd = 18.8$).

These findings suggest that her inspiratory and expiratory muscle strength was more than four standard deviations below the mean, representing profound respiratory muscle weakness. Overall, testing revealed substantial deficits in the respiratory mechanism, particularly in isolated, effort dependent tasks, though respiratory performance appeared adequate for some isolated speech tasks, connected speech, and performance of ADLs. Regarding the later, for example, she was not observed to have shortness of breath or other evidence of respiratory difficulty when ambulating 100 feet or more. The finding of such profound respiratory weakness in an effort dependent task such as MIP and MEP testing is difficult to reconcile with her overall physical performance. Such severely decreased respiratory strength, for example, would be expected to result in speech signs that were not present, such as severely decreased speech loudness and the use of short phrases to allow for frequent respiration. Additionally, deficits in the respiratory mechanism in patients with FAS have not, to our knowledge, previously been reported.

### *6.2. Larynx*

No dysphonia was present in connected speech, though an elevated fundamental frequency (i.e., habitual pitch) was present. During isolated laryngeal testing, a high-pitched, tremulous, and breathy dysphonia was present. Pitch range was variable. Similar to testing of the respiratory mechanism, performance was more disordered on isolated tasks than in connected speech. Laryngeal involvement is not typical in patients with FAS.

### *6.3. Velopharynx*

Velopharyngeal assessment suggested a slight resonance imbalance perceived as mild, variable hypernasality. Isolated tested of velopharyngeal performance appeared consistent with her connected speech performance. Hypernasality or other resonance abnormalities are not commonly reported in organic FAS.

### *6.4. Orofacial mechanism*

Severe right-sided lingual deviation with protrusion was present during oral motor examination. During speech diadochokinesis, the patient was asked to produced “puh”, “tuh”, and “kuh” as rapidly, precisely, and evenly as possible. Her productions were irregular and distorted, and the rate of speech decreased with increased length of production. Speech sound distortions including substitutions, prolongations, omissions, and insertions were evident in connected speech. Vowels were perceived to be more distorted than consonants overall. Tongue bulb testing of lingual strength with the IOPI revealed tongue weakness greater than two standard deviations below the mean [53]. Many articulatory deficits present in this patient's speech are commonly reported in patients with FAS.

### 6.5. Prosody

Multiple prosodic abnormalities were present. Rate of speech was slow. Articulation rates in syllables/sec were obtained from the oral reading of the Grandfather Passage and from a brief sample of spontaneous speech. The procedure employed by Tsao and Weismer [54] to assess oral reading rate was utilized in that pause times equal to or longer than 150 ms were deducted from speaking time. Also, for those phonetic contexts within a sentence in which a word with a final stop sound was followed immediately by a word with an initial stop sound (e.g., "ancient black," "frock coat"), a pause had to be longer than 300 ms to be deducted (55). To measure articulation rate in spontaneous speech, pause times longer than 250 ms were deducted from total speaking time and time spent producing interjections (e.g., "uh," "um,") were not included in the total time. Mean articulation rate during oral reading was 3.40 syllables/sec ($sd = 0.53$). Rate of speech within the 11 individual sentences in the Grandfather Passage ranged from 2.08–4.12 syllables/sec. Tsao and Weismer reported a mean of 3.72 syllables/sec for the 15% of their participants who demonstrated the slowest oral reading articulation rates. Compared to these data, the speaker in the present study evidenced an oral reading articulation rate that was more than one standard deviation below the mean of their slowest group. The FAS speaker's articulation rate in spontaneous speech was somewhat faster. The overall mean rate based on 186 spoken syllables was 4.05 syllables/sec. The sample contained 20 runs of speech uninterrupted by 250 ms or longer pauses or interjections. The mean articulation rate/run was 3.86 syllables/sec ($sd = 0.66$). These runs of speech ranged from 2–22 syllables in length and the articulation rates ranged from 2.39–4.85 syllables/sec. There was a significant correlation between length of run in syllables and articulation rate ($r = 0.740$, $df = 20$, $p < 0.001$) in that longer runs of speech were spoken at a faster articulation rates. These spontaneous speech rates are at the low end of ranges reported in the literature [56,57].

Intonation and stress were both aberrant. Intonation was characterized by unusual, excess pitch variability, while stress was characterized by reduced distinctions between stressed and unstressed syllables. Overall naturalness of speech was found to be severely affected due to its overall very unusual profile of features.

### 6.6. Speech assessment

Motor speech examination revealed more distributed involvement of the speech mechanism than is commonly reported in FAS. Speech signs in FAS are almost invariably related to deficits in articulation and prosody, while this patient also presented with marked involvement of respiration and phonation. Resonance was also affected, though less severely. Many of the patient's speech signs, especially those uncommonly reported in FAS, were only revealed or were magnified in isolated testing, suggesting effort-dependent variability. Connected speech performance was much more consistent with previous descriptions of FAS and emphasized speech signs related to articulatory and prosodic deficits such as sound distortions, substitutions, prolongations, omissions, and insertions, more prominently in vowels than consonants; disordered rate, intonation, stress, and speech naturalness; and decreased intelligibility. More unusually in FAS, mild and variable hypernasality was also present, as well as an elevated fundamental frequency.

## 7. Interventions

### 7.1. Medical therapy

Patient was treated over a multiyear period for chronic daily headaches with migraine features that were reported of sudden and unprovoked onset. She was treated with a range of neuroleptic and psychotropic medication towards the management of affective disturbance and headache pain. More specifically, by the time she was evaluated for biofeedback, she had been unsuccessfully treated with topiramate, amitriptyline, duloxetine, and sumatriptan for her headache. At the time of the psychological evaluation as part of her entry into the biofeedback program, she was at the end of an unsuccessful trial of duloxetine 60mg/day. Her mood and headache pains reportedly remained unaffected. During neurological management subsequent to the referral for biofeedback, she reported that behavioral techniques and pain coping skills training were elevating her mood and producing stability in her headache pains. In addition to headaches, she complained of diffuse joint pain. Thyroid profile, sedimentation rate, and rheumatoid factor evaluation were all checked and found to be in the normal range.

### *7.2. Pain management intervention*

Behavioral interventions for pain management included biofeedback, pain coping skills training, and progressive muscle relaxation training [58,59]. Pain systematically increased from a 4/10 at the first session to a 10/10 by the sixth session, with a focus on her cognitive and affective responses. By the sixth session, she was grimacing, exhibiting a slowed gait, and reporting significantly more pain sites, including her hip. She was described by her therapists as affectively "flat" and minimally responsive to issues unrelated to her physical functioning. She noted that many of her relationships had been altered, but attributed this to her pain and physical limitations. Sessions seven through 13 continued with aggressive behavioral interventions for her chronic pain, though symptom relief was not obtained. The patient was subsequently referred to another headache clinic in her local area.

### *7.3. Speech therapy*

Following her motor speech examination, she was offered a behavioral treatment program targeting her speech sound errors. During her first and only treatment session, the patient demonstrated greater than expected difficulty improving the accuracy of her speech sounds, even with the production of vowels in isolation with maximal modeling and cueing from the clinician. The patient did not return for her next visit. When she was called for follow-up, she indicated that she was satisfied with her current speech and did not desire further intervention to address her speech changes.

## 8. Discussion

Duffy [60] defines the clinical features of FAS as variability in the perceived accent by listeners due to changes in vowels (e.g., distortions, prolongations, insertions, omissions), consonants (e.g., voice, manner, and place distortions; substitutions), and prosody (e.g., inappropriate pitch contours, altered stress, reduced rate). Such speech characteristics indicate prominent involvement of articulation and prosody, producing the impression of a foreign accent to listeners. Rarely does the speech pattern match the non-native speech encountered in a specific language; thus, the term pseudoforeign accent is also appropriate. By any measure, FAS is a rarely encountered communication disorder, with Haley and colleagues [29] estimating the presence of about 50 cases in the literature. We present a case of FAS secondary to conversion disorder based on multidisciplinary assessment and treatment. Our patient was a 39-year-old African-American female who presented with FAS and chronic daily headache. She reported physical discomfort, lacked a clear functional purpose to her symptoms, and denied emotional distress.

Published cases of FAS associated with psychiatric disease are less common but have been reported with increasing regularity over the last decade and have in many ways expanded the definition of what constitutes FAS. Reeves, Burke, and Parker [25] and Reeves and Horton [26] described a total of four patients with psychosis who exhibited signs of FAS which correlated in severity to the severity of their psychotic signs and whose speech gradually returned to baseline with improvement of their psychosis. Gurd and colleagues [61] reported a case of FAS with an unclear etiology in a 47-year-old female who presented with FAS one month following the sudden onset of aphasia and headache. The onset of a French sounding accent was followed shortly by onset of a "bizarre" gait. Although the patient had several small lesions on imaging, the authors "doubted whether all of the patient's symptoms could be accounted for organically" (p. 715). Verhoeven and colleagues [28] described an instance of probable conversion disorder involving a 51-year-old woman who experienced a "psychotrauma" when she was nearly involved in accident while riding her bicycle. She developed FAS and a progressive gait disturbance over the course of the following eight years. Most recently, Haley et al. [29] reported a case of FAS associated with conversion disorder in a 36-year-old female with intermittent episodes of neurological symptoms, such as increased speech and gait disturbances, sensory changes, and weakness. Between acute exacerbations typically lasting two to four days, the patient's accent was considered milder.

Based on review of the available literature and our own clinical experience, we suggest that patients with non-organic FAS exhibit a different clinical course and exhibit different findings on examination. In the case of our patient, her clinical course was very atypical compared the findings reported in patients with FAS secondary to neurologic lesions. For example, she reported waking up one morning with a severe headache that confined her to bed. Two or three days later, she awoke speaking with a foreign accent that she only noticed when others commented on the change. She reported mutism occurred during this period to her speech pathologist and psychologist, but never reported this symptom to her ED physicians or neurologist. Two months later, her persistent headache and speech changes finally caused her to seek medical attention in the ED. Her right-sided sensory loss and abnormal gait did not appear for several more months. This course is clearly very atypical for a patient with FAS due to organic etiology such as CVA or TBI. Additionally, she never demonstrated a lesion of any sort with multiple imaging studies over several years.

Reports identifying conversion disorder in FAS are uncommon so it is difficult to isolate particular features which may differentiate organic versus nonorganic FAS. However, two reports of conversion disorder associated with FAS in the literature [28,29] also presented with unusual gait, similar to the present case. In the present case, as well as the patient described by Haley and colleagues [29], the bilateral gait abnormalities during relapses were suspicious for psychogenic gait disorders, while in the case described by Verhoeven and colleagues [28], an eight year history of progressive gait disturbance eventually left the patient wheelchair bound. Hemiplegia, with or without facial weakness, may also be encountered in FAS, though recovery or improvement often occurs.

Patients with nonorganic FAS also appear to present with more distributed involvement of the speech mechanism than those with organic FAS. While organic FAS is almost exclusively defined by articulatory and prosodic speech changes, in cases of probable and confirmed non-organic FAS, more distributed involvement may be encountered. Laryngeal involvement in the form of dysphonia appears particularly common in nonorganic FAS [27,61]. In our case, dysphonia was only present in isolated testing. This high-pitched, tremulous vocal quality was not present in connected speech. A similar but more pronounced disconnect was present in assessment of the respiratory mechanism. Although careful auditory-perceptual assessment of respiratory function suggested performance grossly within normal limits for speech production, instrumental strength testing of the respiratory mechanism via digital pressure gauge manometry during maximal inhalation and exhalation (i.e., MIP, MEP) revealed profound respiratory weakness. Such respiratory weakness would have serious implications for pulmonary morbidity and mortality and does not appear compatible with her respiratory function during speech or with other observed physical exertion (e.g., ambulating in clinic).

Conversion disorder is a controversial diagnosis. Patients are often diagnosed with a psychosomatic illness based on the absence of medical data supporting an organic cause [62]. One problematic assumption is that all potential organic causes are known and recognizable. A conversion diagnosis also leaves little room to specify both functional and organic etiologies to a presenting condition [63], and the diagnosis itself typically relies on null or inconsistent findings. While misdiagnosis is a concern with conversion disorder, data from Mace and Trimble [64] reveal that if patients undergo comprehensive neurological exams prior to a diagnosis of conversion disorder, less than 15% were misdiagnosed up to 10 years later. In the present case, we suggest that convergent data from repeated examinations by an appropriate multidisciplinary team further reduces the likelihood of misdiagnosis.

Consistent with this approach, we have presented a case of probable conversion disorder based on convergent data from repeated encounters with a multidisciplinary team comprising multiple specialties including neurology, radiology, neuropsychology, psychiatry, and speech-language pathology. These encounters consistently revealed effort-dependent inconsistencies in the patient's exam results without evidence of an underlying physiological cause. Neuropsychological testing revealed poor effort on several memory and motor tasks, as well as discrepancies between simple and complex procedures that is atypical of neurological disease. Similarly, speech function was often found to be more disturbed in isolated testing than in connected speech. Her responses to psychological questionnaires produced profiles highly consistent with a somatoform disorder due to excessive somatic dysfunction in the absence of reported emotional distress. While conversion disorder remains a controversial diagnosis, errors can be minimized by a thorough neurological exam and follow-up consultations from appropriate disciplines. In our attempts to assess and treat this patient, we collaborated across multiple specialties with independent examinations. Our convergent findings assisted in ruling out an organic etiology in this unusual case of FAS secondary to conversion disorder.

## References

[1] K.M. Kurowski, S.E. Blumstein and M. Alexander, The foreign accent syndrome: a reconsideration, *Brain and Language* **54** (1996), 1–25.

[2] C. Avila, J. Gonzalez, M.-A. Parcet and A. Belloch, Selective alteration of native, but not second language articulation in a patient with foreign accent syndrome, *Neuroreport* **15** (2004), 2267–2270.

[3] S.E. Blumstein, M.P. Alexander, J.H. Ryalss, W. Katz and B. Dworetzky, On the nature of the foreign accent syndrome: a case study, *Brain and Language* **31** (1987), 215–244.

[4] D.H. Christoph, G.R. de Freitas, D.P. dos Santos. M.A.S.D. Lima, A.Q.C. Araujo and A. Carota, Different perceived foreign accents in one patient after prerolandic hematoma, *European Neurology* **52**(2004), 198–201.

[5] T. Coughlan, S. Lawson and D. O'Neill, French without tears? Foreign accent syndrome, *Journal of the Royal Society of Medicine* **97**(2004), 242–243.

[6] J. Fridriksson, J. Ryalls, C. Rorden, P.S. Morgan, M.S, George and G.C. Baylis. Brain damage and cortical compensation in foreign accent syndrome, *Neurocase* **11** (2005), 19–24.

[7] J. Gonzalez-Alvarez, M.A. Parcet-Ibars, C. Avila and D. Geffner-Sclarsky, A rare neurologically originated speech disorder: Foreign accent syndrome, *Revista de Neurologica* **36** (2003), 227–234.

[8] P. Marien and J. Verhoeven, Cerebellar involvement in motor speech planning: some further evidence from foreign accent syndrome, *Folia Phoniatrica et Logopaedica* **59**(2007), 210–217.

[9] P.D. Munson and B. Heilman, Foreign accent syndrome: anatomic, pathophysiologic and psychosocial considerations, *South Dakota Journal of Medicine* **58** (2005), 187–189.

[10] E.J. Roth, K. Fink, L.R. Cherney and K.D. Hall, Reversion to a previously learned foreign accent after stroke, *Archives of Physical Medicine and Rehabilitation* **78** (1997), 550–552.

[11] J. Ryalls and J. Whiteside, An atypical case of Foreign Accent Syndrome, *Clinical Linguists and Phonetics* **20** (2006), 157–162.

[12] G.M. Seliger, G.M. Abrams and A. Horton, Irish brogue after stroke, *Stroke* **23** (1992), 1655–1656.

[13] Y. Takayyyama, M. Sugishita, T. Kido, M. Ogawa and I. Akiguchi, A case of foreign accent syndrome without aphasia caused by a lesion of the left precentral gyrus, *Neurology* **43** (1993), 1361–1363.

[14] T.J. Carbary, J.P. Patterson and P.J. Snyder, Foreign Accent Syndrome following a catastrophic second injury: MRI correlates, linguistic and voice pattern analyses, *Brain and Cognition* **43** (2000), 1–3.

[15] R.J. Edwards, N.K. Patel and I.K. Pople, Foreign accent following brain injury: Syndrome or epiphenomenon, *European Neurology* **53** (2000), 87–91.

[16] E. Hem, A peculiar speech disorder due to bomb injury of the brain, *Tidsskr Nor Laegeforen* **126** (2006), 3311–3313.

[17] M. Lippert-Gruener, U. Weinert, T. Greisbach and C. Wedekind, Foreign accent syndrome following traumatic brain injury, *Brain Injury* **19** (2005), 955–958.

[18] A.E. Aronson, *Clinical Voice Disorders* (*3rd Ed.*). Thieme, New York, 1990.

[19] J.I. Bakker, S. Apeldoorn and L.M. Metz, Foreign accent syndrome in a patient with multiple sclerosis, *The Canadian Journal of Neurological Sciences* **31** (2004), 271–272.

[20] R. Villaverde-Gonzalez, E. Fernandez-Villalbbbba, A. Alias-Linares, J.M. Santos, Foreign language syndrome as a first sign of multiple sclerosis, *Revista de Neurologica* **36** (2003), 1035–1039.

[21] S. Luzzi, G. Viticchi, M. Piccirilli, K. Fabi, M. Pesallaccia, M. Bertolini et al., Foreign accent syndrome as the initial sign of primary progressive aphasia, *Journal of Neurology, Neurosurgery, and Psychiatry* **79** (2008), 79–81.

[22] P. Marien, J. Ver hoeven, S. Engelborghs, S. Rooker, B.A. Pickut and P.P. De Deyn, A role for the cerebellum in motor speech planning: evidence from foreign accent syndrome, *Clinical Neurology and Neurosurgery* **108** (2006), 518–522.

[23] T.J. Abel, A.O. Hebb and D.L. Silbergeld, Cortical stimulation mapping in a patient with foreighn acccent syndrome: Case report, *Clinical Neurology and Neurosurgery* **111** (2009), 97–101.

[24] S. Poulin, J, Macoir, N. Paquet, M. Fossard and L. Gagnon, Psychogenic or neurogenic origin of agrammatism and foreign accent syndrome in a bipolar patient: a case report, *Annals of General Psychiatry* **6** (2007), 1.

[25] R.R. Reeves, R.S. Burke and J.D. Parker, Characteristics of psychotic patients with foreign accent syndrome, *Journal of Neuropsychiatry and Clinical Neurosciences* **19** (2007), 70–76.

[26] R.R. Reeves and J.W. Norton, Foreign accent-like syndrome during psychotic exacerbations, *Neuropsychiatry, Neuropsychology, and Behavioral Neurology* **14** (2001), 135–138.

[27] J. Van Borsel, L. Janssens and P. Santens, Foreign accent syndrome: an organic disorder? *Journal of Communication Disorders* **38** (2005), 421–429.

[28] J. Verhoeven, P. Marien, S. Engelborghs, H. D'Haenen and P. De Deyn, A foreign speech accent in a case of conversion disorder, *Behavioural Neurology* **16** (2005), 225–232.

[29] K.L. Haley, H.L. Roth, N. Helm-Estabrooks and A. Thiessen, Foreign accent syndrome due to conversion disorder: Phonetic analyses and clinical course, *Journal of Neurolinguistics* **23** (2009), 28–43.

[30] M.W. Hayes, S. Graham, P. Heldorf, G. de Moore and J.G. Morris, A video review of the diagnosis of psychogenic gait: Appendix and commentary, *Movement Disorders* **14** (1999), 14–21.

[31] M.S. Okun, R.L. Rodriguez, K.D. Foote and H.H. Fernandez, The "chair test" to aid in the diagnosis of psychogenic gait disorders, *The Neurologist* **13** (2007), 87–91.

[32] D. Wechsler, WAIS-III Administration and Scoring Manual. The Psychological Corporation, San Antonio, TX, 1997.

[33] G. Wilkerson, Wide Range Achievement Test 3: Manual. Wide Range, Inc, Wilmington, DE, 1993.

[34] R.M. Reitan, Trail Making Test: Manual for Administration and Scoring. Reitan Neuropsychological Laboratory, Tucson, AZ, 1992.

[35] G.C. Golden, Stroop Color and Word Test: A Manual for Clinical and Experimental Uses. Stoelting Co., Wood Dale, IL, 1978.

[36] R.M. Ruff, Ruff Figural Fluency Test: Administration Manual. Neuropsychological Resources, San Diego, CA, 1988.

[37] D.C. Delis, J.H. Kramer, E. Kaplan and B.A. Ober, *California Verbal Learning Test* (2nd ed.), Psychological Corporation, San Antonio, TX, 2000.

[38] D. Wechsler, Wechsler Memory Scale-Third Edition. Psychological Corporation, San Antonio, TX, 1997.

[39] R.H.B. Benedict, Brief Visuospatial Memory Test-Revised. Psychological Assessment Resources, Inc., Odessa, FL, 1997.

[40] E.H. Kaplan, H. Goodglass and S. Weintraub, *The Boston Naming Test*. Lea and Febiger, Philadelphia, PA, 1983.

[41] A.L. Benton, K.D. Hamsher and A.B. Sivan, *Multilingual Aphasia Examination*. AJA Associates, Iowa City, IA, 1994.

[42] P. Green, Green's Word Memory Test for Microsoft Windows: User's Manual. Green's Publications Inc., Edmonton, Canada, 2005.

[43] T.N. Tombaugh, *Test of Memory Malingering Manual*, Multi-Health Systems, New York, NY, 1996.

[44] A.T. Beck, R.A. Steer and G.K. Brown, *Beck Depression Inventory-II Manual*, Psychological Corporation, San Antonio, TX, 1996.

[45] G. Arnold, K.B. Boone, P. Lu, A. Dean, J. Wen, S. Nitch and S. McPherson, Sensitivity and specificity of finger tapping test scores for the detection of suspect effort, *The Clinical Neuropsychologist* **19** (2005), 105–120.

[46] J.N. Butcher, W.G. Dahlstrom, J.R. Graham, A. Tellegen and B. Kaemmer, The Minnesota Multiphasic Personality Inventory-2 (MMPI-2): Manual for Administration and scoring. University of Minnesota Press, Minneapolis, MN, 1989.

[47] P.T. Costa, Jr. and R.R. McCrae, The NEO Personality Inventory Manual, Psychological Assessment Resources, Odessa, FL, 1985.

[48] S.R. Briggs, The optimal level of measurement for personality constructs, in: *Personality psychology: Recent Trends and Emerging Directions*, D.M. Buss and N. Cantor, eds, Springer–Verlag, New York, NY, 1989, pp. 246–260.

[49] L.R. Derogatis, SCL-(90)-R: Administration, scoring, and procedures manual (2nd ed.), Clinical Psychometric Research, Baltimore, MD, 1983.

[50] C.D. Spielberger, R.L. Gorsuch and R.E. Lushene, Manual for the State Trait Anxiety Inventory. Consulting Psychologists Press, Palo Alto, CA, 1970.

[51] R.D. Kent, J.F. Kent and J.C. Rosenbek, Maximum performance tests of speech production, *Journal of Speech and Hearing Disorders* **52** (1987), 367–387.

[52] H.I. Chen and C.S. Kuo, Relationship between respiratory muscle function and age, sex, and other factors, *Journal of Applied Physiology* **66** (1989), 943–948.

[53] H.C. Crow and J.A. Ship, Tongue strength and endurance in different aged individuals, The Journals of Gerontology: Series A, Biological sciences and medical sciences, **51** (1996), M247–50.

[54] Y.C. Tsao and G. Weismer, Interspeaker variation in habitual speaking rate: evidence for a neuromuscular component, *Journal of Speech, Language, and Hearing Research* **40** (1997), 858–866.

[55] P. Flipsen, Articulation rate and speech-sound normalization failure, *Journal of Speech, Language, and Hearing Research* **46** (2003), 724–737.

[56] F. Goldman-Eisler, Psycholinguistics: Experiments in Spontaneous Speech, Academic Press, London, 1968.

[57] B. Lindblom, Economy of speech gestures, in: *The Production of Speech* P.F. MacNeilage, ed., Springer–Verlag, New York, NY, 1983, pp. 217–246.

[58] C.L. Edwards, M. Feliu, S. Johnson, W. Webster, G.G. Bennett, D. Bishop et al., The application of cognitive-behavioral techniques for the management of complicated and persistent gout pain, *Ciencias de la Conducta* **20** (2005), 89–116.

[59] F.J. Keefe, M. Jacobs and C. Edwards, Persistent pain: Cognitive-behavioral approaches to assessment and treatment, *Seminars in Anesthesia, Perioperative Medicine and Pain* **16** (1997), 117–126.

[60] J.R. Duffy, *Motor Speech Disorders: Substrates, Differential Diagnosis, and Management, 2nd ed., ition*). Elsevier Mosby, St. Louis, 2005.

[61] J.M. Gurd, J.S. Coleman, A. Costello and J.C. Marsh, Organic or functional? A new case of foreign accent syndrome, *Cortex* **37** (2001), 715–718.

[62] C.V. Ford and D.G. Folks, Conversion disorders: An overview, *Psychosomatics* **26** (1985), 371–374, 380–383.

[63] J. Stone, M. Zeidler and M. Sharpe, Misdiagnosis of conversion disorder, *American Journal of Psychiatry* **160** (2003), 391; author reply 391–2.

[64] C.J. Mace and M.R. Trimble, Ten-year prognosis of conversion disorder, *Britich Journal of Psychiatry* **169** (1996), 282–288.
